# Supplementary material for: Effects of cytochrome P450 2B6 and constitutive androstane receptor genetic variation on Efavirenz plasma concentrations among HIV patients in Kenya
Source: PLoS One. 2022 Mar 2;17(3):e0260872. doi: 10.1371/journal.pone.0260872 (PMC8890732; doi:10.1371/journal.pone.0260872)
Supplement: S1 Fig — 15582C>T, 516G>T, 785A>G, 983T>C, 21563C>T and 18492C>T significantly influence EFV plasma concentration (p<0.05) but not 1459C>T and CAR 540C>T. (PDF) [file pone.0260872.s001.pdf]

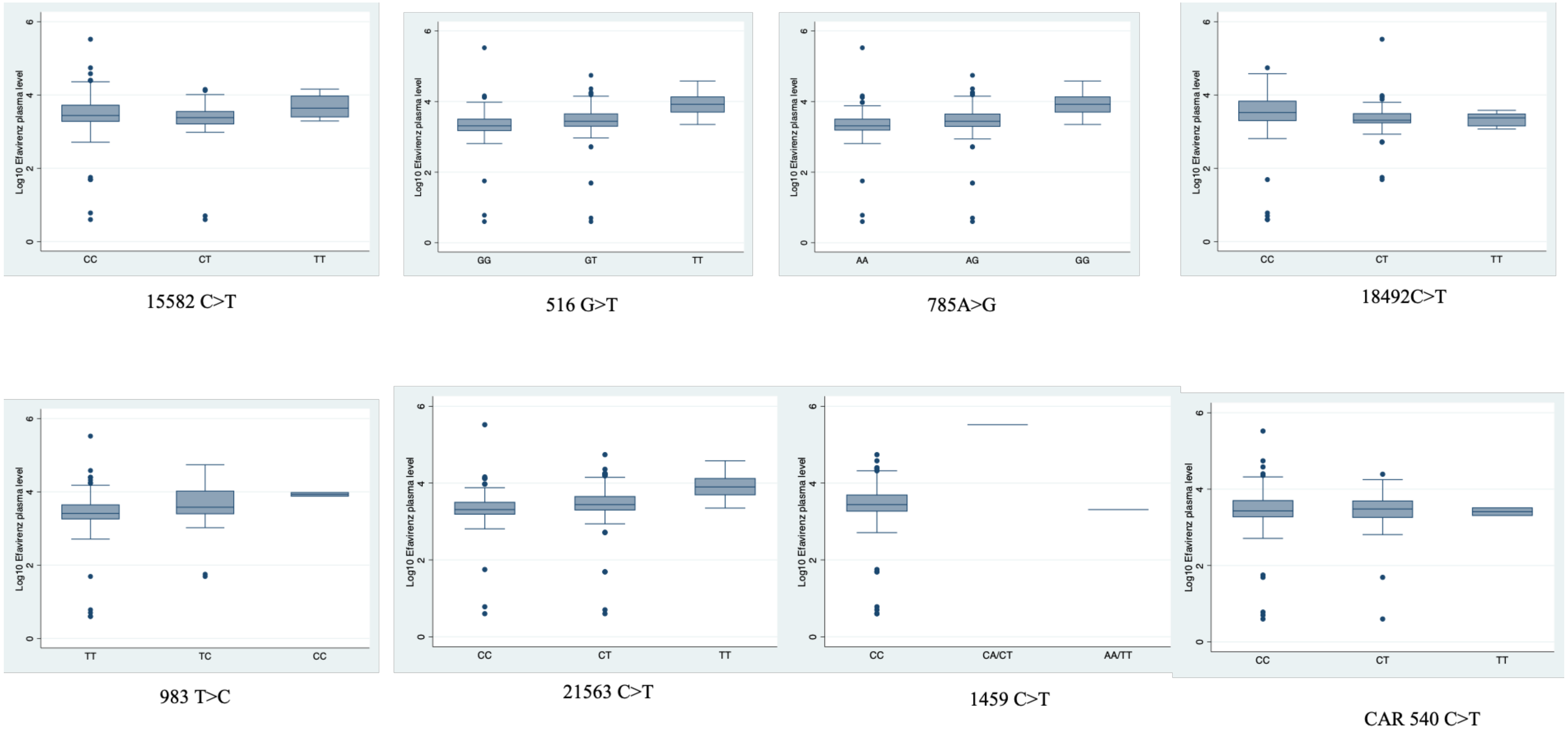

S1 Fig. The differences in $\log_{10}$-transformed EFV plasma concentrations by genotypes of 7 CYP2B6 and 1 CAR SNPs. 15582C>T, 516G>T, 785A>G, 983T>C, 21563C>T and 18492C>T significantly influence EFV plasma concentration ($p<0.05$) but not 1459C>T and CAR 540C>T.
