# Supplementary material for: Effects of cytochrome P450 2B6 and constitutive androstane receptor genetic variation on Efavirenz plasma concentrations among HIV patients in Kenya
Source: PLoS One. 2022 Mar 2;17(3):e0260872. doi: 10.1371/journal.pone.0260872 (PMC8890732; doi:10.1371/journal.pone.0260872)
Supplement: S2 Fig — Dark red squares: strong evidence of LD, dark yellow/orange squares: uninformative, light yellow squares: strong evidence of recombination. SNP1-15582C>T; SNP2 - 516G>T; SNP3 - 785A>G; SNP4-18492C>T; SNP5- 983T>C; SNP6-21563C>T; SNP7- 1459C>T and SNP8—CAR 540C>T. (PDF) [file pone.0260872.s002.pdf]

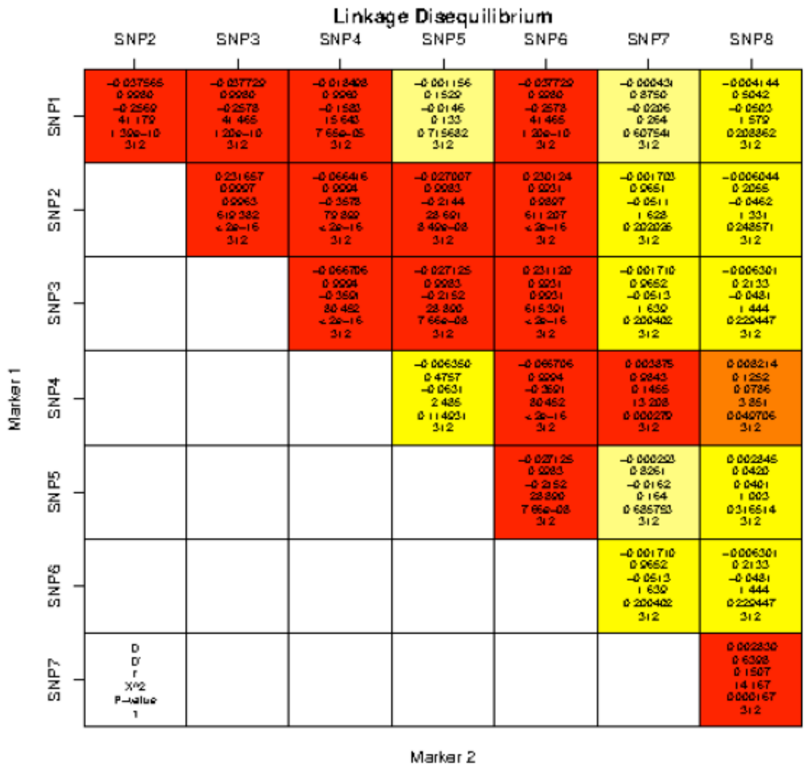

S2 Fig. Linkage disequilibrium analysis of 7 SNPs of CYP2B6 and 1 CAR. Dark red squares: strong evidence of LD, dark yellow/orange squares: uninformative, light yellow squares: strong evidence of recombination. SNP1-15582C>T; SNP2 - 516G>T; SNP3 - 785A>G; SNP4-18492C>T; SNP5- 983T>C; SNP6-21563C>T; SNP7- 1459C>T and SNP8 - CAR 540C>T.
