# Supplementary material for: Effects of cytochrome P450 2B6 and constitutive androstane receptor genetic variation on Efavirenz plasma concentrations among HIV patients in Kenya
Source: PLoS One. 2022 Mar 2;17(3):e0260872. doi: 10.1371/journal.pone.0260872 (PMC8890732; doi:10.1371/journal.pone.0260872)
Supplement: Attachment — Submitted filename: Response to Reviewers.docx [file pone.0260872.s004.docx]

Isabelle Chemin, PhD
Academic Editor
PLOS ONE

**RE: Response to reviewers’ comments for the manuscript PONE-D-21-00146
Effects of cytochrome P450 2B6 and constitutive androstane receptor genetic variation on Efavirenz plasma concentrations among HIV patients in Kenya**

Find a point by point responses to the issued raised by reviewers

**Comment:** Improved in bringing precisions in the material and methods, throughout the whole text, and clarifications in figures and results should be done.

***Response****: The methodology has been improved and the protocol used also deposited in the laboratory protocols in protocols.io to enhance the reproducibility of your results.*

**Comment:** Please ensure that your manuscript meets PLOS ONE's style requirements, including those for file naming.

***Response****: The format of the manuscript has been adjusted in line with the PLOS ONE style templates can be found at*

https://journals.plos.org/plosone/s/file?id=wjVg/PLOSOne_formatting_sample_main_body.pdf and

[https://journals.plos.org/plosone/s/file?id=ba62/PLOSOne_formatting_sample_title_authors_affiliations.pdf](about:blank)

**Comment:** During our internal evaluation of the manuscript, we found significant text overlap between your submission and the following previously published works, some of which you are an author.

https://www.futuremedicine.com/doi/10.2217/pgs.11.160 (Introduction, paragraph 1, sentences 1-2)
https://www.zora.uzh.ch/id/eprint/56011/1/Efavirenz-CYP2B6-CAR-JAntimicrobChemother2011-ZORA.pdf (Introduction, paragraph 1, sentences 4-5)
https://academic.oup.com/jac/article/66/9/2092/771232  (Introduction, paragraph 1, sentence 6 & paragraph 2, sentence 1)
https://pericles.pericles-prod.literatumonline.com/doi/10.1002/mgg3.598 (Introduction, paragraph 2, sentence 2)
https://journals.plos.org/plosone/article?id=10.1371%2Fjournal.pone.0130583 (Introduction, paragraph 2, sentences 3-4 & Discussion, paragraph 2, sentence 3)
https://journals.plos.org/plosone/article?id=10.1371/journal.pone.0130583 (Discussion, paragraph 1, sentence 1)
https://journals.sagepub.com/doi/10.1177/2050312118780861 (Discussion, paragraph 4, sentence 2)
https://f1000research.com/articles/9-363 (Discussion, paragraph 6, sentence 2 & paragraph 10, sentence 2)
https://ascpt.onlinelibrary.wiley.com/doi/full/10.1002/cpt.1477 (Discussion, paragraph 6, sentence 2)
https://www.nature.com/articles/s41598-018-23350-1 (Discussion, paragraph 11, sentence 3)
https://insights.ovid.com/british-clinical-pharmacology/bjcpha/2012/12/000/pharmacogenetic-markers-cyp2b6-associated/13/00002256 (Discussion, paragraph 11, sentence 4)

***Response:*** *in the revised manuscript all the duplicated text have been rephrased. The revised manuscripts also show the advances in knowledge from previous work*

**Comment**: In your Data Availability statement, you have not specified where the minimal data set underlying the results described in your manuscript can be found. PLOS defines a study's minimal data set as the underlying data used to reach the conclusions drawn in the manuscript and any additional data required to replicate the reported study findings in their entirety. All PLOS journals require that the minimal data set be made fully available. For more information about our data policy, please see <http://journals.plos.org/plosone/s/data-availability>.

***Response:*** *Data used in this study have been supplied as Supporting Information files*

Upon re-submitting your revised manuscript, please upload your study’s minimal underlying data set as either Supporting Information files or to a stable, public repository and include the relevant URLs, DOIs, or accession numbers within your revised cover letter. For a list of acceptable repositories, please see http://journals.plos.org/plosone/s/data-availability#loc-recommended-repositories. Any potentially identifying patient information must be fully anonymized.

**Comment**. Please ensure that you refer to Figure 2 in your text and check your figure legend numbering in the manuscript as, if accepted, production will need this reference to link the reader to each figure.

***Response****: The figure and table numberings and reference in the text have been redone in line with manuscript guidelines*

**Comment:** Your ethics statement should only appear in the Methods section of your manuscript. If your ethics statement is written in any section besides the Methods, please delete it from any other section.

***Response:*** *The ethics statement has been inserted in the Methods section only*

Reviewers' comments

Reviewer's Responses to Questions

**Comments to the Author**

1. Is the manuscript technically sound, and do the data support the conclusions?

The manuscript must describe a technically sound piece of scientific research with data that supports the conclusions. Experiments must have been conducted rigorously, with appropriate controls, replication, and sample sizes. The conclusions must be drawn appropriately based on the data presented.

Reviewer #1: Yes

2. Has the statistical analysis been performed appropriately and rigorously?

Reviewer #1: No

3. Have the authors made all data underlying the findings in their manuscript fully available?

The [PLOS Data policy](http://www.plosone.org/static/policies.action#sharing) requires authors to make all data underlying the findings described in their manuscript fully available without restriction, with rare exception (please refer to the Data Availability Statement in the manuscript PDF file). The data should be provided as part of the manuscript or its supporting information, or deposited to a public repository. For example, in addition to summary statistics, the data points behind means, medians and variance measures should be available. If there are restrictions on publicly sharing data—e.g. participant privacy or use of data from a third party—those must be specified.

Reviewer #1: Yes

4. Is the manuscript presented in an intelligible fashion and written in standard English?

PLOS ONE does not copyedit accepted manuscripts, so the language in submitted articles must be clear, correct, and unambiguous. Any typographical or grammatical errors should be corrected at revision, so please note any specific errors here.

Reviewer #1: No

**5. Review Comments to the Author**

Please use the space provided to explain your answers to the questions above. You may also include additional comments for the author, including concerns about dual publication, research ethics, or publication ethics. (Please upload your review as an attachment if it exceeds 20,000 characters)

Reviewer #1: The authors report associations of genetic variation of CYP2B6 (13 variants) and constitutive androstane receptor (CAR) (one variant) on efavirenz (EFV) plasma concentration among 312 HIV Kenyan patients who were on efavirenz-based first-line ART regimen. The authors confirm published associations between CYP2B6 variants and efavirenz exposure.

**Major comments**
**Comment:** The manuscript is poorly communicated - needs a careful editing of several typos, repetitive statements and inaccurate references

***Response:*** *The manuscript has been edited to improve on language and eliminate typos, repetitive statement and inaccurate references*

**Comment:** The way the data has been analyzed is somewhat inadequate (see below) and it needs careful check by a statistician. Moreover, it would be advisable to go over the tables and figures carefully to make sure the values presented are correct and make sense.

***Response:*** *Data re-analyzed and tables and figures rechecked and improved and or corrected.*

**Comment:** The basis for the calculation of metabolic score is not well justified and the data derived from it is suspect.

***Response****: The whole section of metabolic score has been dropped from the manuscript*

**Comment:** Too much redundancy in the discussion section and this part could be significantly shortened without losing the major findings.

**Response:** Discussion section edited appropriately to improve on the flow
**Other comments**
Abstract:
Comment 1. What does (p>0.0001) mean?

**Response. The p value has been corrected**

**Comment** 2. There seems a discrepancy in the proportion of patients outside the efavirenz concentration (22% versus 36.2%). Please correct this discrepancy.

***Response****. Discrepancy corrected*

**Comment** 3. How was “High number of patients (17.9%) had an inferred ultra-rapid metabolic phenotype” determined.

***Response****: Metabolic section deleted from the document*

**Introduction:**
**Comments** 4. First paragraph (2nd sentence) has multiple problems. First, Wyen et al., 2013 is given as a reference for CYP2A6-mediated efavirenz metabolism, which was not included in the reference list. Second, I am not sure that is the best reference for CYP2A6-mediated metabolism (PMID: 20335270 probably better). Third, I am not sure why CYP2B6 is repeated in that sentence. I think, that sentence needs revision by saying for example such as…. Efavirenz 7-hydroxylation by CYP2B6 and direct N-glucuronidation by UGT2B7 represent minor metabolic pathways. Please use the original references for CYP2A6 and UGT2B7.
5. First paragraph: UGT2B is probably type and shows up out of the blue? I would imagine this to be UGT2B7 and that is why they need first to provide metabolism by UGT2B7 as suggested above.
6. First paragraph: Wyen et al., 2013 is given in the text, but Wyen et al., 2011 is given in the reference list. Please address this inconsistency
7. Second paragraph: CYP2B6 allele nomenclature. www.cypalleles.ki.se/cyp2b has been retired. Use the PharmVar web page for this reference

***Repose:*** *The introduction has been rewritten to eliminate reference mismatch and those missing. All the original references have been included and those missing included. The inconsistency in references also addressed. The reference listing CYP2B6 alleles has been changed from CYP2B6 allele nomenclature.* [*www.cypalleles.ki.se/cyp2b*](http://www.cypalleles.ki.se/cyp2b) *to Pharmacogene Variation Consortium. Available at* [*https://www.pharmvar.org/gene/CYP2B6*](https://www.pharmvar.org/gene/CYP2B6) *accessed July, 2021*

**Study design and settings:**

Comment 8. The citation Ngayo et al., (2016) is not listed in the reference list.

***Response:*** *This reference was cited as number 24 in the reference list except that the author’s full names were written as Musa Otieno Ngayo. This has since been adjusted accordingly*
**Data collection:**

**Comment 9.** Was the whole blood sample for genotyping and drug bassay collected in the same tube or different tubes? Please specify

***Response:*** *The blood collection tubes have been specified as follows “Blood samples (10 mL) at 12–16 h post ARV uptake was collected into three blood tube as follows: EDTA anticoagulant tube for immunological testing and CYP2B6 and CAR genotyping. Serum separating tube for clinical chemistry while Lithium heparin tube for HIV viral load and EFV plasma level quantification*

LC/MS/MS assay:

**Comment 10.** Provide assay dynamics (intra- and interday, LOQ, linear range etc). Also what was the internal standard used

***Response:*** *the assay dynamics have been presented as solutions, selectivity, Recovery and Linearity and accuracy and precision in the method section*

Results section:
**Comment 11a.** Table 2: It is intriguing that the HIV-RNA >1000copies/mL and mutations was more prominent in the normal efavirenz concentration range than the lower range.

***Response.*** *The distribution of viral load remains as stated above. Data has been provided for confirmation*

**Comment 11b** It is stated that non-adherence rate was 23.1%. Was this taken into account among the low, normal and high efavirenz concentration group? It may also help to include the non-adherence rate among the groups in Table 2.

***Response****. The non-adherence rate for the past 30 days was reported as 207/312 (66.3) which has been compared across the EFV therapeutic ranges. This has been included in the table 2*

**Comment 12**. What was the basis for calculating the metabolic scores? This calculation seems to have major flaws. First, it assumes the functional consequences of each SNP are the same. This assumption is wrong. In terms of in vivo functional consequences, the SNPs can be categorized as follows from high impact to low impact: CYP2B6 983T>C (almost null allele) > CYP2B6 516G>T >> CYP2B6 15582C>T and CYP2B6 1459C>T (functional). CYP2B6 785A>G is in linkage disequilibrium with CYP2B6 516G>T forming CYP2B6*6, but when it occur alone (*4) is associated with increased activity. Although CYP2B6 18492C>T is associated with lower efavirenz concentrations, how is that possible to assign 18492 TT = −2; 18492 CT = −1; 18492 CC = 0. I think this type of functional classification and the subsequent designation of metabolic status (Figure 2) and results associated is unlikely to be valid. How inferred ultra-rapid is also assigned remains unclear. Overall, this paragraph is very confusing. There are also typos (1459TT, 1459TC and 1459TT).

***Response****: All the section of Metabolic score and inferred phonotypes has been deleted from the manuscript*

**Comment 13**. Table 4 and the texts in the body of the manuscript. The 95 % CI are the same as the difference. Something wrong?

***Response****: The data has been re-analyzed by a statistician and discrepancies corrected both in the text and in Table*

**Comment 14**. The values listed in Table 5, particularly the Beta Coefficients, seem odd. First, the direction of effect in the unadjusted and adjusted does not always match (e.g., unadjusted beta for 21563C>T 1577 while adjusted is -8512.8). Second, the values of the beta are huge and unrealistic. It may be prudent to check the statistical analysis.

***Response****: The data has been re-analyzed by a statistician and discrepancies corrected both in the text and in Table*

**Discussions:**
**Comment 15**. First paragraph (1st and 2nd sentence): the link between CYP2B6 genetics and efavirenz concentrations has been established in many populations of African descent. I would start with a sentence something like “This is the first analysis in Kenyans with sufficient sample size and expanded variants in CYP2B6 variants, etc”. Also it could not be comprehensive for CAR genetics when only one SNP was tested.

***Response****: The sentence has been edited to reflect the true picture*

**Comment 16**. 2nd paragraph: provide appropriate references to the last sentence

***Response****: Reference by Haas ety al., 2004 cited*

**Comment 17**. Third paragraph (about metabolic score): remove this paragraph and Figure 1 unless providing adequate justification for the categorization (see comments #9 above).

**Comment 18**. Fourth paragraph: the authors state that “In agreement with our study, a significant proportion of patients with virologic failure (33.3%) had suboptimal EFV plasma concentration compared to 10% of the patients with EFV plasma concentration within therapeutic range”. I do not think this sentence is consistent with findings in Table 2 where the prevalence is much higher in the group with normal range

***Response****; The sentence corrected in line with the table*

**Comment** 19. Fifth paragraph. Were patients with TB and on anti-TB included in this study, which might have created the discrepancy with the other studies?

**Response**; TB patients or those on anti-TB not included in the study

C**omment** 20. Six paragraph: the lack of PK interaction between efavirenz and NRIs has been well established and does not add new information (can be deleted).

***Response****; This paragraph deleted*

**Comment** 21. Typo (two figure 1). I think Figure 1 can go to supplemental files (data are already displayed in Table 2). The other figure 1 (which should be figure 2) could be deleted.

***Response****. The figures corrected accordingly and figure 1included in the supplementary section*

**Comment** 22. Figure 3 is difficult to read and can go to supplemental file.

***Response****. Figure 3 taken to supplementary section as S2 Fig*

6. PLOS authors have the option to publish the peer review history of their article ([what does this mean?](https://journals.plos.org/plosone/s/editorial-and-peer-review-process#loc-peer-review-history)). If published, this will include your full peer review and any attached files.


If you choose “no”, your identity will remain anonymous but your review may still be made public.


**Do you want your identity to be public for this peer review?** For information about this choice, including consent withdrawal, please see our [Privacy Policy](https://www.plos.org/privacy-policy).

Thank you for your valuable comments which we have incorporated the suggested changes and we hope that manuscript meets the requirements of the journal.

Looking forward for a positive response from you

Kind regards,

**Dr. Musa Otieno Ngayo**

Centre for Microbiology Research

KEMRI

Enclosed is our manuscript
