# Supplementary material for: Effects of cytochrome P450 2B6 and constitutive androstane receptor genetic variation on Efavirenz plasma concentrations among HIV patients in Kenya
Source: PLoS One. 2022 Mar 2;17(3):e0260872. doi: 10.1371/journal.pone.0260872 (PMC8890732; doi:10.1371/journal.pone.0260872)
Supplement: Attachment — Submitted filename: Reviewer comments 2_21_2021.docx [file pone.0260872.s003.docx]

The authors report associations of genetic variation of CYP2B6 (13 variants) and constitutive androstane receptor (CAR) (one variant) on efavirenz (EFV) plasma concentration among 312 HIV Kenyan patients who were on efavirenz-based first-line ART regimen. The authors confirm published associations between CYP2B6 variants and efavirenz exposure.

**Major comments**

- The manuscript is poorly communicated - needs a careful editing of several typos, repetitive statements and inaccurate references
- The way the data has been analyzed is somewhat inadequate (see below) and it needs careful check by a statistician. Moreover, it would be advisable to go over the tables and figures carefully to make sure the values presented are correct and make sense.
- The basis for the calculation of metabolic score is not well justified and the data derived from it is suspect.
- Too much redundancy in the discussion section and this part could be significantly shortened without losing the major findings.

**Other comments**

**Abstract:**

1. What does (p>0.0001) mean?
2. There seems a discrepancy in the proportion of patients outside the efavirenz concentration (22% versus 36.2%). Please correct this discrepancy.
3. How was “High number of patients (17.9%) had an inferred ultra-rapid metabolic phenotype” determined.

**Introduction:**

4. First paragraph (2^nd^ sentence) has multiple problems. First, Wyen et al., 2013 is given as a reference for CYP2A6-mediated efavirenz metabolism, which was not included in the reference list. Second, I am not sure that is the best reference for CYP2A6-mediated metabolism (PMID: 20335270 probably better). Third, I am not sure why CYP2B6 is repeated in that sentence. I think, that sentence needs revision by saying for example such as….Efavirenz 7-hydroxylation by CYP2B6 and direct N-glucuronidation by UGT2B7 represent minor metabolic pathways. Please use the original references for CYP2A6 and UGT2B7.
5. First paragraph: UGT2B is probably type and shows up out of the blue? I would imagine this to be UGT2B7 and that is why they need first to provide metabolism by UGT2B7 as suggested above.
6. First paragraph: Wyen et al., 2013 is given in the text, but Wyen et al., 2011 is given in the reference list. Please address this inconsistency
7. Second paragraph: CYP2B6 allele nomenclature. [www.cypalleles.ki.se/cyp2b](http://www.cypalleles.ki.se/cyp2b) has been retired. Use the PharmVar web page for this reference

**Study design and settings:**

8. The citation Ngayo et al., (2016) is not listed in the reference list

Data collection:

9. Was the whole blood sample for genotyping and drug bassay collected in the same tube or different tubes? Please specify

**LC/MS/MS assay:**

10. Provide assay dynamics (intra- and interday, LOQ, linear range etc). Also what was the internal standard used

**Results section:**

11. Table 2: It is intriguing that the HIV-RNA >1000copies/mL and mutations was more prominent in the normal efavirenz concentration range than the lower range. It is stated that non-adherence rate was 23.1%. Was this taken into account among the low, normal and high efavirenz concentration group? It may also help to include the non-adherence rate among the groups in Table 2.
12. What was the basis for calculating the metabolic scores?. This calculation seems to have major flaws. First, it assumes the functional consequences of each SNP are the same. This assumption is wrong. In terms of in vivo functional consequences, the SNPs can be categorized as follows from high impact to low impact: CYP2B6 983T>C (almost null allele) > CYP2B6 516G>T >> CYP2B6 15582C>T and CYP2B6 1459C>T (functional). CYP2B6 785A>G is in linkage disequilibrium with CYP2B6 516G>T forming CYP2B6*6, but when it occur alone (*4) is associated with increased activity. Although CYP2B6 18492C>T is associated with lower efavirenz concentrations, how is that possible to assign 18492 TT = −2; 18492 CT = −1; 18492 CC = 0. I think this type of functional classification and the subsequent designation of metabolic status (Figure 2) and results associated is unlikely to be valid. How inferred ultra-rapid is also assigned remains unclear. Overall, this paragraph is very confusing. There are also typos (1459TT, 1459TC and 1459TT).
13. Table 4 and the texts in the body of the manuscript. The 95 % CI are the same as the difference. Something wrong?
14. The values listed in Table 5, particularly the Beta Coefficients, seem odd. First, the direction of effect in the unadjusted and adjusted does not always match (e.g., unadjusted beta for 21563C>T 1577 while adjusted is -8512.8). Second, the values of the beta are huge and unrealistic. It may be prudent to check the statistical analysis.

**Discussions:**

15. First paragraph (1^st^ and 2^nd^ sentence): the link between CYP2B6 genetics and efavirenz concentrations has been established in many populations of African descent. I would start with a sentence something like “This is the first analysis in Kenyans with sufficient sample size and expanded variants in CYP2B6 variants, etc”. Also it could not be comprehensive for CAR genetics when only one SNP was tested.
16. 2^nd^ paragraph: provide appropriate references to the last sentence
17. Third paragraph (about metabolic score): remove this paragraph and Figure 1 unless providing adequate justification for the categorization (see comments #9 above).
18. Fourth paragraph: the authors state that “In agreement with our study, a significant proportion of patients with virologic failure (33.3%) had suboptimal EFV plasma concentration compared to 10% of the patients with EFV plasma concentration within therapeutic range”. I do not think this sentence is consistent with findings in Table 2 where the prevalence is much higher in the group with normal range
19. Fifth paragraph. Were patients with TB and on anti-TB included in this study, which might have created the discrepancy with the other studies?
20. Six paragraph: the lack of PK interaction between efavirenz and NRIs has been well established and does not add new information (can be deleted).
21. Typo (two figure 1). I think Figure 1 can go to supplemental files (data are already displayed in Table 2). The other figure 1 (which should be figure 2) could be deleted.
22. Figure 3 is difficult to read and can go to supplemental file.
